# Near-Fatal Aspiration in a Child With Pierre Robin Sequence and Aero-Digestive Disorder: A Case Report

**DOI:** 10.7759/cureus.66106

**Published:** 2024-08-04

**Authors:** Gayathri Balasubramaniam, Subashini Vichili Mohan, Balaji Ramamurthy

**Affiliations:** 1 Anaesthesiology, SRM Institute of Science and Technology, Chengalpattu, IND

**Keywords:** postoperative pulmonary complications, eras protocol, difficult airway, palatoplasty, aerodigestive disorders

## Abstract

Pierre Robin sequence (PRS) presents significant challenges in airway management and postoperative care, especially in infants undergoing cleft palate repair. The most critical task for paediatric anaesthetists is securing the airway. The presence of aero-digestive disorders makes postoperative care equally challenging, which is often underemphasised. This report describes the management of a 17-month-old male child with PRS and a partial cleft palate who aspirated postoperatively following palatoplasty. Prompt intervention with nebulised bronchodilators, oxygen therapy, and intravenous antibiotics led to significant clinical improvement. The case underscores the necessity of developing standardised guidelines for managing children post-surgery.

## Introduction

Children with the Pierre Robin sequence (PRS) are at increased risk for airway obstruction, dysphagia, and aspiration due to micrognathia (undersized lower jaw) and glossoptosis (displacement of the tongue), which position the tongue toward or even against the posterior pharyngeal wall [1]. Ensuring airway patency remains paramount before any definitive treatment in PRS [2]. Feeding problems and failure to thrive (FTT) are frequently encountered. Various factors contribute to these issues, including low socioeconomic status, swallowing dysfunction, abnormal sucking quality, repeated micro-aspiration, and gastro-oesophageal reflux [3-5]. Interventions for children with PRS should thus be evaluated based on their effectiveness in improving respiratory problems and promoting weight gain [1].

Aero-digestive disorders, encompassing a complex interplay of dysfunction in the airway, swallowing mechanism, and digestive system, pose significant challenges for infants, particularly those with compromised anatomy [6]. The overall incidence of PRS affects 1 in 8,500 to 1 in 14,000 newborns per year [7]. However, the overall incidence of PRS with aero-digestive complications arises in 1 in 20,000 births [8]. The peri-operative physician must ensure that the child’s overall health is optimal in children with aero-digestive disorders.

The upper digestive tract (oesophagus), pulmonary tract (trachea, bronchi, and lungs), and airway (pharynx and larynx) are all parts of the aero-digestive tract. This system helps with breathing and swallowing, which calls for a coordinated and intricate series of motions to safeguard the airway when eating and drinking and ensure adequate airflow for speaking. The larynx and hyoid bone are positioned higher in newborns than in adults, and the soft palate (velum) resides near the epiglottis. The quick 'suck-swallow-breathe' sequence required for breastfeeding is supported by this anatomical configuration. In order to protect the airway, sucking, swallowing, and breathing must be coordinated properly. However, infants with cleft palate, particularly those with velopharyngeal dysfunction, often experience aero-pharyngeal difficulties. They struggle to maintain the 'suck-swallow-breathe' sequence, which leads to breathing challenges during feeding. Typical symptoms include coughing, choking, gagging, difficulty breathing, and oxygen desaturation following aspiration encountered during feeding.

After surgery, the separation of the oral and nasal cavities and the creation of the uvula result in a significant reduction of oral space. Children need time to unlearn and relearn the swallow-breathe sequence postoperatively. Factors such as pain and oedema make this process even more challenging. Some children, lacking the inherent neurohumoral intelligence, take longer to relearn the sequence and re-establish feeding after surgery.

This case report presents a near-fatal aspiration event in a child with PRS, emphasising the critical need for monitoring during feeding and meticulous airway management. It sheds light on the complexities of airway management in a child with PRS and aero-digestive disorders, highlighting the potential for life-threatening complications.

## Case presentation

**Figure 1:**
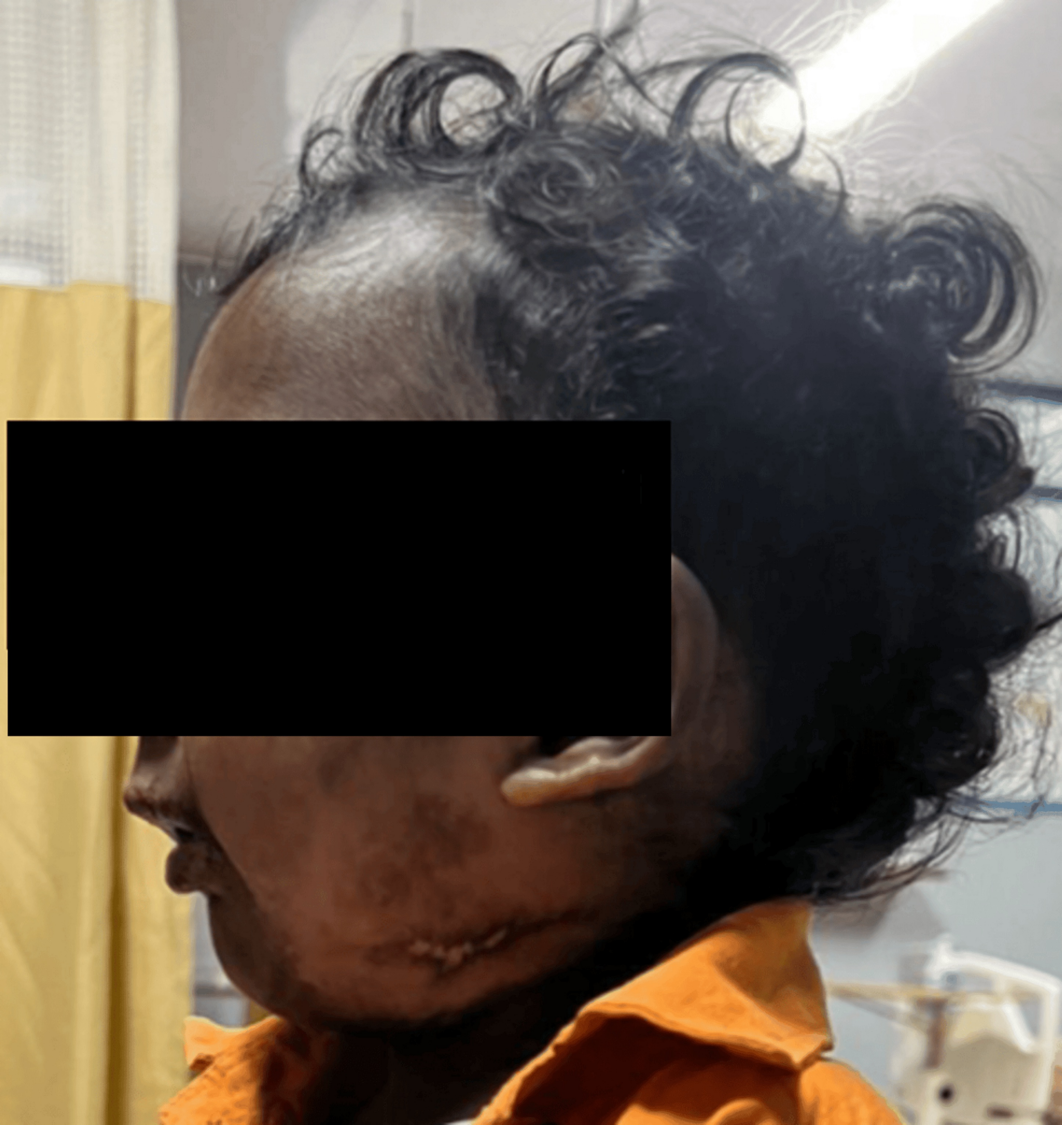
A 17-month-old male child with PRS PRS: Pierre Robin sequence

**Figure 2:**
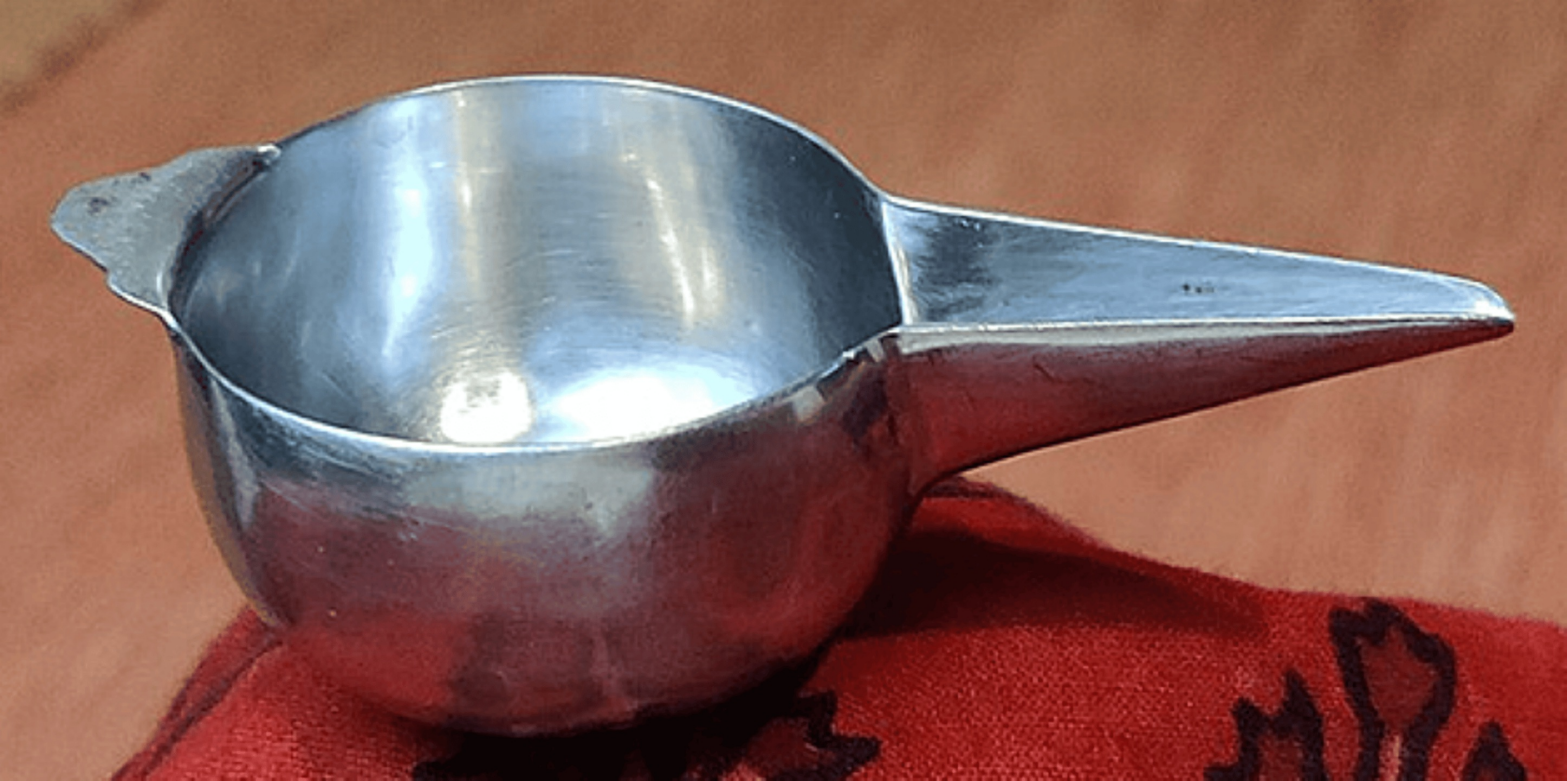
Paaladai

**Figure 3:**
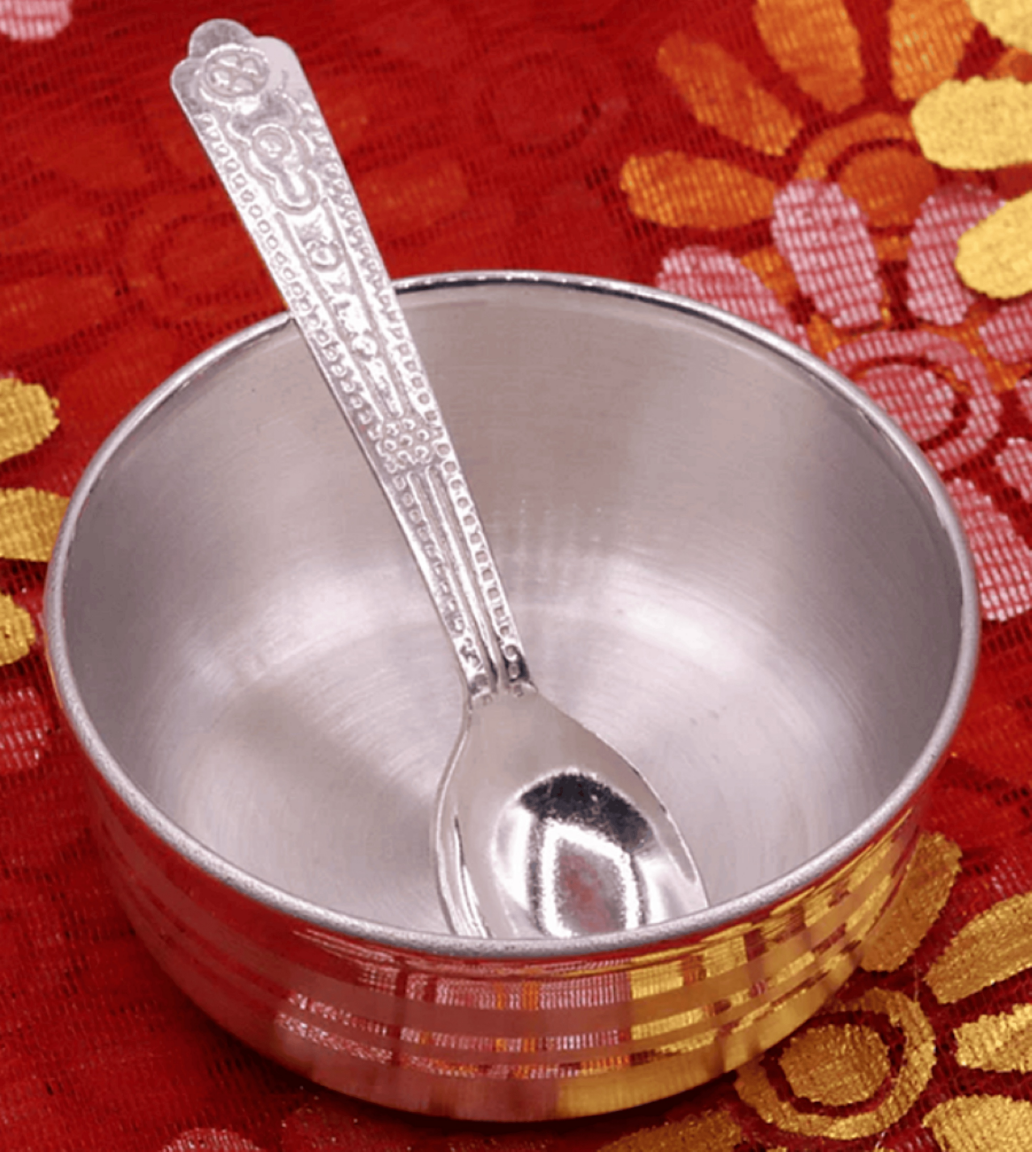
Feeding bowl

A 17-month-old male child weighing 9.6 kg presented with a partial cleft of the secondary palate and features consistent with PRS (Figure 1). The child was scheduled for primary cleft palate repair under general anaesthesia and was assessed by the American Society of Anesthesiologists-III (ASA-III). The mother gave a typical history of coughing and gagging during feeding from birth. Postoperatively, the child underwent early supervised oral feeding (milk) as per the Enhanced Recovery After Surgery (ERAS) protocol, which was well tolerated. The mother was trained to feed the child with milk using a bowl and spoon or paaladai (Figures 2-3).

On postoperative day 1 (POD 1), the child aspirated milk in the ward, leading to respiratory distress and desaturation (SpO_2_: 91%). He presented with coughing, irritability, and subcostal and substernal retractions. He was transferred to the Post-Operative Anesthesia Care Unit (PACU) with oxygen support. Auscultation revealed bilateral wheezing. He received nebulisation with albuterol, alternating with ipratropium bromide, for three cycles. The child responded well to the treatment, and within six hours, his vitals returned to normal. The chest X-ray was normal.

Oral feeds were discontinued for a day, and the child was maintained on intravenous fluids (Ringer's lactate) at 40 mL/hr, along with an intravenous injection of amoxicillin and potassium clavulanate 300 mg three times a day. On POD 2, the child was reassessed and showed clinical improvement, with resolution of noisy breathing, wheezing, and cough. However, substernal retraction persisted. The paediatrician recommended restarting supervised oral feeds and continuing nebulised albuterol and ipratropium bromide for another day.

## Discussion

Aero-digestive disorders in children disrupt the coordinated functions of the airway, oesophagus, and upper gastrointestinal tract, potentially leading to chronic respiratory issues from the aspiration of food or liquids into the lungs. Children with cleft conditions experience these challenges prior to surgical repair, and it's noted that issues may persist post-surgery, entailing ongoing attention. Effective management requires a multidisciplinary team, including anaesthesiologists, surgeons, and speech-language pathologists (SLPs), to guide and develop comprehensive treatment strategies.

Lee et al. compared the incidence of aspiration pneumonia in newborns with complete and incomplete cleft palates. Of the 100 children analysed, 35 (35%) had aspiration pneumonia. Similar incidences of the disease were seen in those with complete and incomplete cleft palates (27 of 70 (39%) vs. 8 of 30 (27%), p = 0.36). The lower left lobe (11 of 35 (31%)) was the most frequently affected by pneumonia, followed by the right upper and lower lobes [9].

Another study by Muhammad et al. from Pakistan reported that the incidence of aspiration in children with a cleft palate was as high as 64.53%, with 19.85% of patients experiencing recurrent pneumonia. The mortality rate due to complications associated with this disorder was 7.09% [10].

Fathy and Attia conducted a study to evaluate the needs of mothers with infants who have a cleft lip and/or palate. The study involved 50 mothers of infants with these conditions. Data were collected using a structured questionnaire and an assessment form to evaluate the infants' physical and psychological needs. The findings revealed that 75% of the mothers did not know how to prevent aspiration in their infants, which could lead to pneumonia. The study concluded that some mothers struggled to manage the health problems associated with caring for their infants with orofacial defects. It recommended addressing the developmental needs and health issues of infants with a cleft lip and/or palate and providing mothers with education, especially on feeding techniques for a child with a cleft [11].

Eridani-Ball and Brimble conducted a case study on the care of an infant born with a cleft palate. They concluded that care should emphasise family-centred approaches and multidisciplinary teamwork, with children's nurses playing a pivotal role [12].

In a cross-sectional study, Turlapati et al. found that only 21% of mothers reported an improvement in their infants' feeding ability after cleft palate surgery. The study noted that factors such as the type of cleft, timing of surgery, and presence of other health issues significantly influence recovery to near-normal function. Early closure of the soft palate is recommended, as it helps generate adequate intraoral pressure, thereby enhancing the baby's sucking ability [13]. In a similar study conducted by de Vries et al., 79% of parents reported an improvement in feeding after surgery, while feeding problems persisted in the remaining 21% of children [14].

Paediatric ERAS protocols offer a comprehensive, evidence-based approach to maximise a child's recuperation following surgery. Preoperative patient education, shorter preoperative fasting intervals, minimally invasive surgical techniques, multimodal analgesia, early supervised oral feedings, early mobilisation, and improved respiratory recovery are the mainstays of ERAS. Recent advancements in cleft palate surgeries have introduced the ERAS protocol. Studies indicate a substantial 49% reduction in postoperative opioid use and a 45% shorter time to achieving the first oral intake. Key elements of the protocol include administering gabapentinoids, performing maxillary nerve blocks, and utilising non-opioid medications like acetaminophen and ibuprofen. No significant differences in the PACU, hospital length of stay, or respiratory recovery were noted [15].

Aero-digestive centres are dedicated to providing a multidisciplinary approach to address the diverse issues faced by these children. Chronic pulmonary aspiration is a common consequence in patients with aero-digestive disease. Swallowing assessments using techniques such as video fluoroscopic swallow study (VFSS) and fibreoptic endoscopic evaluation of swallowing (FEES) are commonly employed. To thoroughly assess upper airway dynamics and the functional anatomy of swallowing, otolaryngologists and SLPs usually collaborate while conducting FEES studies. The goal of aero-digestive programs is to provide this complex and medically vulnerable group with reliable, productive, cost-effective, results-oriented, patient-centred, and family-focused treatment [16].

## Conclusions

This report underscores the importance of comprehensive postoperative care involving a multidisciplinary team to address the needs of these children. Optimal nursing protocols tailored to these patients are vital. Equally crucial is educating parents about these protocols to enhance the overall success and safety of the postoperative period, thus minimising the risk of airway and respiratory complications associated with cleft repair surgeries.
